# An Automated Procedure for Evaluating Song Imitation

**DOI:** 10.1371/journal.pone.0096484

**Published:** 2014-05-08

**Authors:** Yael Mandelblat-Cerf, Michale S. Fee

**Affiliations:** McGovern Institute for Brain Research, Department of Brain and Cognitive Sciences, Massachusetts Institute of Technology, Cambridge, Massachusetts, United States of America; Utrecht University, Netherlands

## Abstract

Songbirds have emerged as an excellent model system to understand the neural basis of vocal and motor learning. Like humans, songbirds learn to imitate the vocalizations of their parents or other conspecific “tutors.” Young songbirds learn by comparing their own vocalizations to the memory of their tutor song, slowly improving until over the course of several weeks they can achieve an excellent imitation of the tutor. Because of the slow progression of vocal learning, and the large amounts of singing generated, automated algorithms for quantifying vocal imitation have become increasingly important for studying the mechanisms underlying this process. However, methodologies for quantifying song imitation are complicated by the highly variable songs of either juvenile birds or those that learn poorly because of experimental manipulations. Here we present a method for the evaluation of song imitation that incorporates two innovations: First, an automated procedure for selecting pupil song segments, and, second, a new algorithm, implemented in Matlab, for computing both song acoustic and sequence similarity. We tested our procedure using zebra finch song and determined a set of acoustic features for which the algorithm optimally differentiates between similar and non-similar songs.

## Introduction

Songbirds learn to sing by imitating the vocalizations of their parents or other conspecific birds to which they are exposed at a young age [1], [2], [3]. Song production and learning are under the control of complex social and behavioral factors [4], [5] and are mediated by cortical and basal ganglia circuits with a striking homology to similar circuits underlying motor learning in the mammalian brain [6], [7]. Thus, songbirds have emerged as a tractable model system to study the neural mechanisms underlying the generation and learning of complex behaviors acquired through practice, such as speech and musical performance [8].

The most commonly used songbird for laboratory studies of vocal learning is the zebra finch, which produce bouts of singing lasting from 1–5 seconds. The song of adult zebra finches consists of a sequence of 3–7 distinct song syllables called a motif. The order of the syllables within the motif, as well as the acoustic structure within each syllable, is typically produced in a fairly stereotyped fashion across song renditions.

Like all songbirds, zebra finches learn to sing in a series of stages, beginning with an exposure to a tutor song while still in the nest. During this stage, the young bird forms a memory of the tutor song, called a song template [3]. At around 30 days post hatch (dph), zebra finches begin to babble, producing highly variable vocalizations called subsong. Over the course of 4–6 weeks of practice, during the plastic song stage, the song of a young zebra finch gradually becomes more structured and more similar to the tutor song [9]. Vocal variability gradually decreases [10] until, at sexual maturity (80–90 dph) the song achieves the highly stereotyped structure of adult song.

The mechanisms underlying vocal learning are not yet fully understood. Vocal learning and maintenance in songbirds is dramatically disrupted by deafening or other hearing impairments [11], [12], [13], [14], leading to the view that vocal learning requires the integration of auditory feedback with vocal/motor commands [15]. According to one model of vocal learning, a comparison of the bird's own song with the song template provides an ‘error signal’ that can be used to reinforce song variations that were a better match to the template [7], [16], [17], [18]. Another model suggests that auditory feedback may be used during babbling to learn the relation between motor commands and vocal output. Such an ‘inverse model’ could then be used to reconstruct the sequence of motor commands needed to produce a good match to the song template [19], [20]. To test models such as these, it is necessary to study the effects of different behavioral, neuronal or other manipulations on song learning or song production [21], [22], [23], [24], [25]_ENREF_26.

Early efforts at quantifying song imitation were made using visual inspection of song spectrograms [4], [24]. However, the difficulty of assessing song similarity visually, as well as the need for a uniform metric across research labs, spurred the development of computerized methods of song comparison. In one approach [26], the song spectrum is represented at each moment by a small number of spectral features, and the similarity of two sounds is measured as the Euclidean distance in this low-dimensional space. Song imitation is assessed by, first, manually selecting a segment of pupil song and a segment of tutor song. Then, using the feature-based distance metric, regions of high similarity between the segments of pupil and tutor songs are identified, and the results are aggregated into a global measure of acoustic similarity and sequence similarity. Typically, the song segments chosen for such a comparison are song motifs of both the pupil and tutor birds. This approach to the analysis of song similarity is the basis of a widely-used software package (Sound Analysis Pro, SAP).

In the process of using SAP to analyze the extent to which young birds had imitated their tutors, we discovered several challenges. Young birds, as well as those that had undergone experimental manipulations, produced songs that were less stereotyped than normal adult songs, and contained vocal elements that could not be easily identified as components of a motif. As a result, it was unclear exactly which parts of a song bout to include in the analysis, raising concerns about possible inconsistencies and experimenter bias in the selection process. Here we have developed a well-specified automated procedure for selecting segments of pupil song, thus reducing the potential for experimenter bias.

Existing algorithms for evaluating the acoustic and sequence similarity of pupil and tutor song depend on the segmentation of song into syllables and silent gaps. The variability of juvenile songs makes such segmentation highly unreliable, and motivated us to develop a new algorithm for evaluating song similarity that treats pupil song as a continuous stream of sound, without segmenting it into syllables and gaps. We have tested this algorithm with different sets of acoustic features to determine which ones maximize the contrast between similarity scores of different renditions of the same bird and similarity scores between different birds. We also compared this measure of contrast to that achieved by SAP software for a database of adult zebra finch songs, and examined the performance of this algorithm on quantifying the development of song imitation in juvenile birds. The algorithm was implemented in Matlab, and is made available in Supplementary Materials (File S1).

## Results

**Figure 1:**
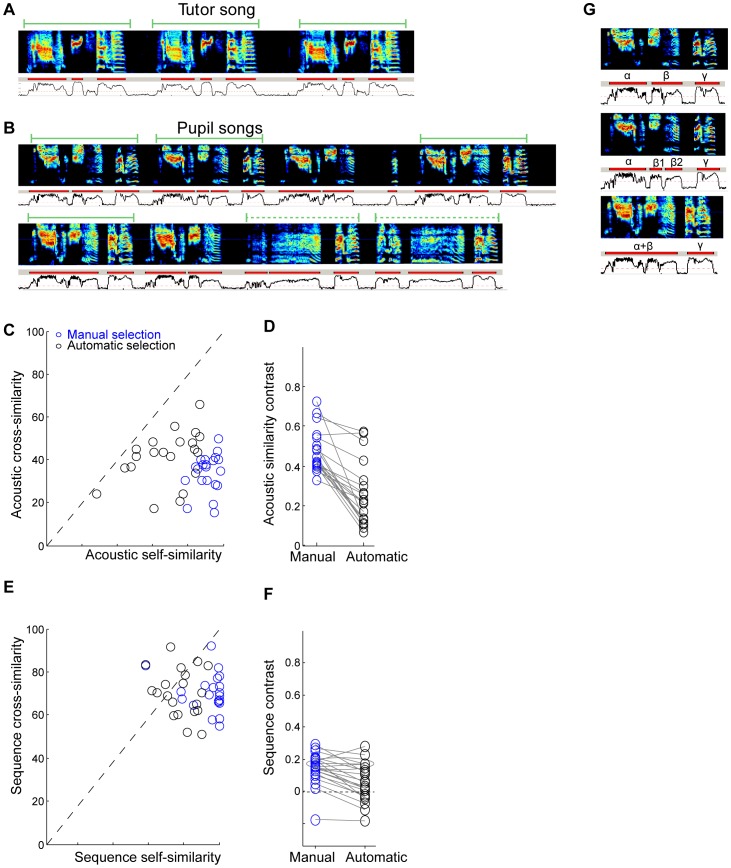
Selection of tutor and pupil songs for similarity analysis. A) Example of tutor song showing stereotyped motifs. B) Examples of pupil song bouts, showing variable song structure including motifs and other irregular vocal elements. Some segments of pupil song are more similar to the tutor motif than others. Green bars represent apparent motifs. C–F) The effect of manual (blue) versus automatic (black) selection of song segments on similarity analysis (computed with Sound Analysis Pro, SAP). C) The songs of each bird are compared to other songs of the same bird (self-similarity) or to songs of other adult birds in the colony (cross-similarity). Each point in (C) plots the self-similarity vs cross-similarity score for one bird based on the acoustic similarity of the songs. D) Contrast between self- and cross-similarity, computed for acoustic similarity scores. Each point is one bird. E) Sequence self-similarity versus sequence cross-similarity. F) Contrast between self- and cross-similarity, computed for sequence similarity scores. Each line in figures D,F connects results from the same birds, carried out either by manually or automatically selecting song segments. G) Examples of inconsistent segmentation of song into syllables and silent gaps. For panels A-B, G: top, song spectrogram; middle, segmentation of the song into syllables (red bars); bottom, sound amplitude (log power).

As noted above, one important concern is that analyzing song imitation typically involves manual selection of the segments of pupil song to compare with the tutor motif. If the pupil has reached adulthood and produces a stereotyped song motif, then it is usually straightforward to hand select a representative selection of motifs on which to carry out the comparison with the tutor motif (Figure 1A). However, in pupil birds with limited or variable imitation of the tutor motif, different song sections may be more or less similar to the tutor (Figure 1B, solid and dashed green bars, respectively). The outcome of the comparison to the tutor song will depend on the method used to select pupil song segments for comparison. This clearly raises the possibility that comparisons between experimental and control groups could be affected by experimenter bias, and complicates comparisons of experimental outcomes across different laboratories.

To illustrate the extent to which hand-selection of song material can impact the outcome of a similarity measurement, we compared an analysis run on manually-selected song segments with an analysis run, by the same algorithm, on randomly-selected song segments. We assessed the outcomes by quantifying the contrast between measurements of similarity between different songs of the same bird (self-similarity) and measurements of song similarity between different adult birds in our colony (cross-similarity). The contrast is defined as the difference between the self-similarity score and cross-similarity score divided by the sum of these two scores (see Methods). Multiple song motifs were manually selected from 21 adult birds in our colony and a similarity analysis was carried out with Sound Analysis Pro (batch comparison). As expected, we observed high acoustic similarity between songs produced by the same bird, and low acoustic similarity between songs of different birds (SAP similarity scores of 91% and 34%, respectively, Figure 1C,D).

We then ran an automated procedure that extracted motif-length song segments from bouts of song. This was done by splitting bouts into non-overlapping segments, each having the duration of a song motif. The extracted segments were confirmed to include only song vocalizations (see Methods), and were then loaded into SAP to carry out the same similarity analysis used for the manually-selected song segments. This selection process resulted in a significantly lower acoustic similarity between songs from the same bird (self-similarity: 68% as compared to 91%, Wilcoxon rank-sum test, p<10^−4^), and a higher acoustic similarity between songs of different birds (cross-similarity: 41% as compared to 34%, Wilcoxon rank-sum test p = 0.014). Taken together, the contrast between self-similarity and cross-similarity (their difference divided by their sum, see Methods) was reduced by almost a factor of two for automated song selection compared to manual selection (0.25 as compared to 0.46; Figure 1D, Wilcoxon rank-sum test p<10^−4^, paired t-test p<10^−6^; note that the acoustic similarity computed by the SAP algorithm is designed to be insensitive to the sequence of syllables)

We also examined the impact of automated versus manual song selection on measures of song sequence similarity. The contrast between song sequence similarity within each bird and sequence similarity across birds also decreased significantly when the song renditions were automatically selected (Figure 1E,F. contrast 0.14 and 0.06, manual and automatic selection, respectively; Wilcoxon rank-sum test p = 0.011, paired t-test p<10^−4^). Note that the contrast measures for sequence similarity using both manual and automatic selection were significantly smaller than the contrast measures for acoustic similarity (both paired t-test p<10^−4^).

These findings suggest that even bouts of normal adult zebra finches song can contain variations in vocal structure that are not captured when hand-selecting motifs. Indeed, visual inspection of song spectrograms reveal that this arises from a combination of variations in song sequence as well as the presence of song vocal elements (syllables) not part of the canonical song motif. Thus, measures of song similarity will depend strongly on the method of selecting song segments used in the analysis. This effect is likely to be even more pronounced when analyzing young or experimentally-manipulated pupil birds with highly variable songs. Since manual selection inevitably involves subjective decisions on how to handle song variations, we conclude that it is better to use an automated process for selecting segments of pupil song. The most important benefit of using an automatic selection method is that it is precisely specified, and can be consistently applied across all data sets.

Another difficulty in assessing song similarity, imposed by the presence of song variability, is related to the identification of silent gaps between syllables. Clearly, the timing of gaps within the song is a crucial component of song structure, and any measure of song similarity must incorporate gaps. On the other hand, silent gaps naturally have a high mutual similarity because their song spectral features have a characteristic value. Thus a direct comparison of pupil gap and tutor gaps do not meaningfully contribute to a measure of song similarity.

One approach is to segment the songs to syllables and silent gaps, and carry out only syllable-to-syllable comparisons — simply eliminating gaps from the process. However, this approach would fail if the identification of syllables and gaps is unreliable, for example, because of variability in the duration of a short gap. If two adjacent sound sequences are sometimes segmented into one syllable and sometimes segmented into two syllables (Figure 1G), this would result in very different syllable similarity scores. Such unreliability in segmentation could have a particularly deleterious impact on measures of sequence similarity.

To avoid these concerns, the SAP algorithm does not carry out syllable-to-syllable comparisons, but instead detects ‘islands of similarity’ between the two songs. While this approach allows flexibility in capturing similarities in spite of possible merging or splitting of syllables, it also allows different parts of a segmented pupil syllable to be matched to completely different (non-sequential) parts of the tutor song, even if a reasonable match to the complete syllable exists. Therefore, this approach may result in overall overestimation of the similarity, which decreases the contrast between songs that are indeed similar and those that are not. It also reduces the reliability of the sequencing score.

## The Algorithm

We set out to devise an algorithm that measures the similarity between the songs of one bird with potentially highly variable songs (for example a pupil bird) and the song of another bird that has a stereotyped song motif (for example a tutor bird). First we will specify a procedure for selecting segments of tutor and pupil for comparison. Second, we will describe an algorithm to compute the similarity between the selected segments.

### Song selection

It is assumed that tutor songs are sufficiently stereotyped that song imitation can be quantified using a small number of representative song motifs, and that these can be hand selected in an unbiased way by the experimenter. Thus, tutor song motifs were manually selected and segmented to syllables and silent gaps (using our Song-GUI software tool). For tutor birds that had a highly stereotyped song motif, at least 3 motif samples were collected. For tutor birds that had a small number of different ‘versions’ of their motif, 3 samples of each version were collected.

In contrast to the manual selection of tutor song segments, the pupil song segments are selected automatically from within bouts of song by the software. Pupil song is treated as a continuous signal, without segmenting it into syllables and silent gaps. The steps of this procedure are as follows:

First, pupil song bouts were extracted (using Song-GUI), excluding only introductory notes and non-song vocalizations, such as distance calls, that occurred between bouts. In an effort to minimize experimenter bias, we kept continuous streams of vocalization intact in these segments, even if they contain vocal elements that were not easily identifiable as song syllables. The software then automatically partitions extracted pupil song bouts into non-overlapping adjacent segments. Each segment is twice the length of the tutor motif, and as many segments are extracted from the pupil bout as possible. To ensure proper sampling of different time alignments, the first segment is not drawn from the beginning of the bout, but starts at a random time after the bout onset, ranging between zero and the duration of a tutor motif.

### Acoustic and sequence similarity scores

**Figure 2:**
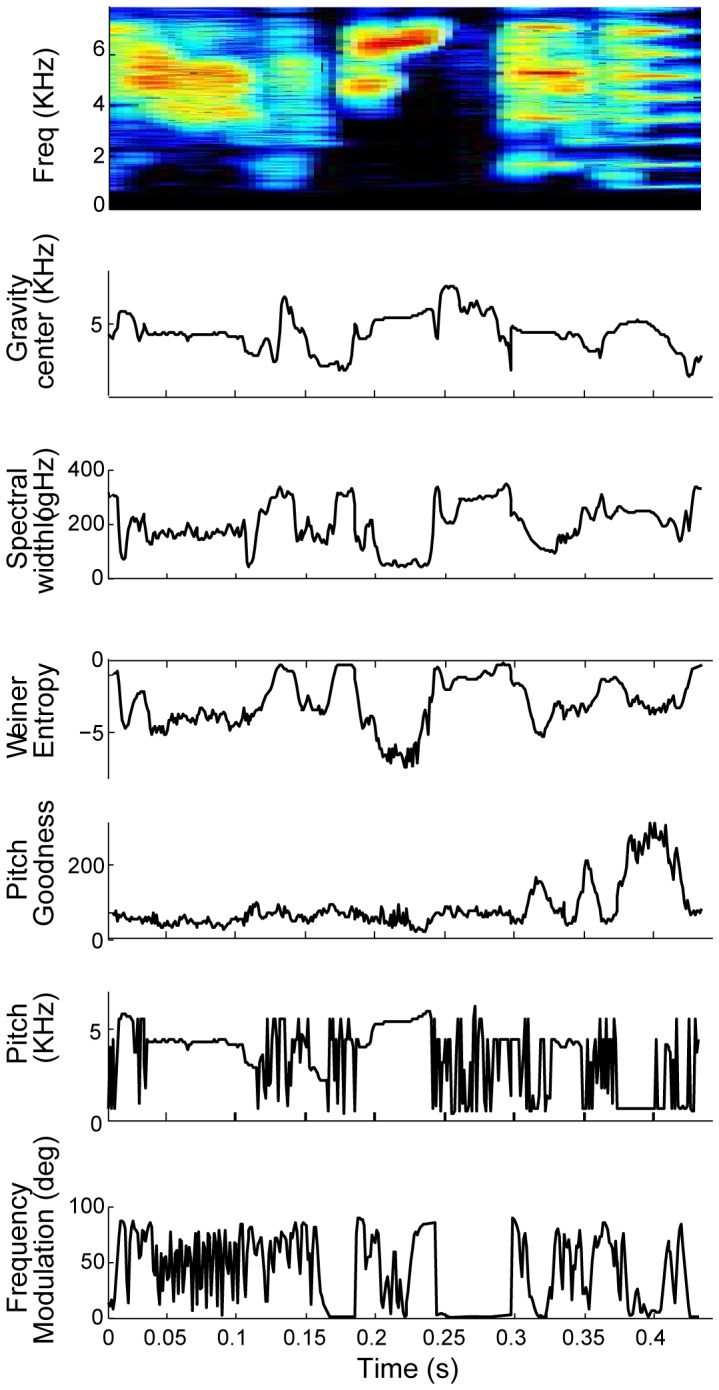
Spectral features for representation of song. We consider the following 6 features for representing song acoustic structure: Top to bottom: gravity center, spectral width, Weiner entropy, pitch goodness frequency modulation and pitch.

To compute acoustic similarity, we adopted the approach of Tchernichovski [26] in representing songs by a small set of spectral features, each of which is computed from the song spectrum computed in short (9 ms) time slices of the sound pressure signal. The spectral features we consider are: Wiener entropy, frequency modulation (FM), pitch, pitch goodness, gravity center, and spectral width (Figure 2, see Methods). Each feature was mean-subtracted and scaled so that the absolute median difference from its mean is one (see Methods). In the following descriptions, the notation *f_1_, f_2_, f_3_, … f_Nf_* represents the set of *N_f_* mean-subtracted and normalized song features. The notation *f_k_(i)* represents the value of the *k^th^* feature at the *i^th^* time point.

### Construction of the similarity matrix

The method of calculating a similarity matrix was adopted from Tchernichovski et al [26]. The detailed steps are as follows: First, we construct a distance matrix in which each bin represents the Euclidean distance in the feature space between time windows in the tutor and pupil songs. Let D (*M*, *N*) be a rectangular matrix where *M* is the number of time points in tutor song and *N* is the number of time points in the pupil song. The distance between the set of features in bin *i* of the tutor song and bin *j* of the pupil song is: 
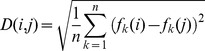



Because the matrix of distances calculated in short time windows is too noisy to reliably identify regions of high song similarity, like Tchernichovski et al, we compute a separate distance matrix containing the weighted average along diagonals of the distance matrix *D*. The RMS average distance is computed along diagonals 25 ms on either side of each point (*i,j*):
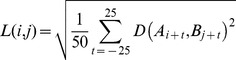



Finally, the distance matrices D and L were transformed into similarity matrices. The approach used by Tchernichovski et al, which we adopt here, is to relate each entry in the distance matrix D or L to the probability of observing that value. This is done as follows: A set of distance matrices were computed for pairwise comparisons of the songs of 10 unrelated adult birds, and the cumulative distribution of distance values in D and L was computed across these birds. The cumulative distributions are stored, and allow us to assign a probability, *P*(D_i,j_) and *P*(L_i,j_), of observing a distance value less than each observed D_i,j_ and L_i,j_ in our actual tutor-pupil song comparison.

Finally, a similarity matrix is computed using the distance matrices D and L, incorporating the probability distributions described above. This is done in two stages: In the first step, regions of similarity are determined from the distance matrix L, and are defined as bins (*i,j*) for which the probability P(L_i,j_) of observing a smaller distance in unrelated songs is less than 0.05 (namely, *P(L_i,j_)<0.05*). In the second step, the value of the similarity matrix *S_i,j_* is assigned as *S_i,j_ = 1-P(D_i,j_)* if the bin i,j is within a region of similarity, otherwise S_i,j_ is set to zero. Thus, the similarity matrix S ends up containing bins of non-zero value only in regions of high similarity (as determined from the L distance matrix). However, the numerical values of S are not determined from L, but are given by *S_i,j_ = 1-P(D_i,j_)*.

### Acoustic similarity score

**Figure 3:**
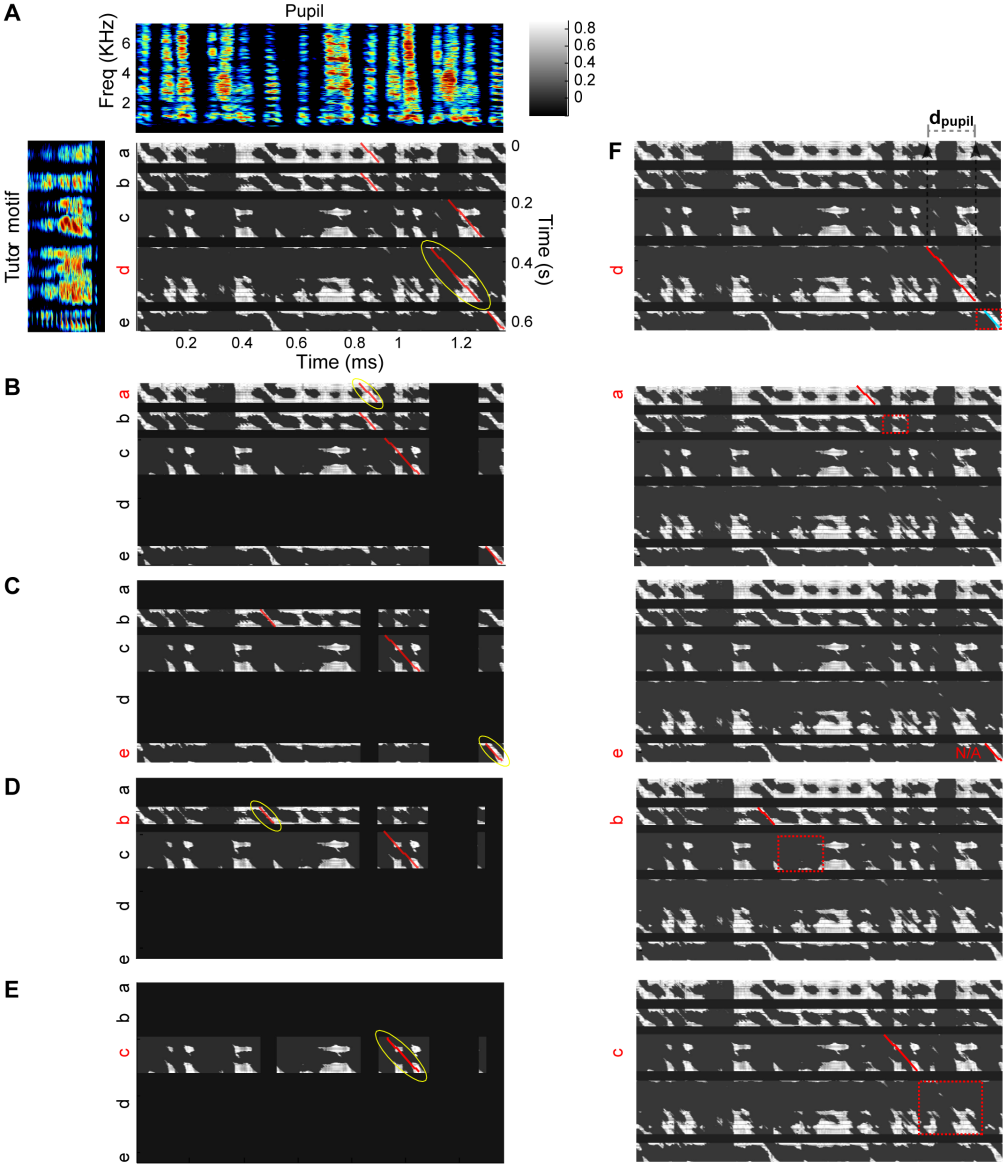
Computation of acoustic and sequence similarity from the similarity matrix. A) Pupil song segment (spectrogram at top), tutor motif (at left) and matrix of similarities between all time points in the two songs. For each of the tutor syllables at left, the red diagonal line represents the best match in the pupil song. The yellow circle marks the diagonal with the highest scores (computed as the integral along the diagonal). This represents the selected best match to that tutor syllable. B–E) The best-matched tutor syllable and section of pupil song are removed from the similarity matrix. B) The best matches to the remaining tutor syllables are recomputed. The yellow circle marks the diagonal with the largest score. C–E) The best-matched tutor syllable and pupil song section are removed from the similarity matrix and the process is reiterated until all tutor syllables have been matched. F) Computation of sequence score. Top panel: For syllable ‘d’ the best matching is shown by a red diagonal and, for illustration, the matched pupil song fragment is denoted here by d_pupil_. The algorithm then measures the similarity between the next tutor syllable (‘e’) and the fragment of pupil song which follows d_pupil_. This area of interest is marked by a dashed red box, below and to the right of the red diagonal. The algorithm finds the highest-scoring diagonal within the area of interest, denoted here by cyan diagonal, and this is the partial sequence score for syllable d. Other panels: partial sequence scores for the other syllables. The overall sequence score is the average over the partial sequence scores for all syllables.

The computation of acoustic similarity score from the similarity matrix treats the tutor syllables as independent entities, while treating the pupil song as a continuous stream, without consideration of its syllable and gap structure. Thus, the similarity matrix is composed of horizontal bands corresponding to tutor syllables. Each band is bounded above and below by regions, corresponding to silent gaps in the tutor song, which are not considered in the analysis. Within this framework, the procedure matches each tutor syllable with a fragment of the pupil song to which it is most similar. The process utilizes a greedy strategy, starting with the tutor syllable that has the best match in the pupil song, and, working iteratively, ends with the syllable that has the worst match (see Figure 3). Note that this process is based on matching individual complete tutor song syllables, rather than the ‘islands’ of similarity used by Tchernichovski et al.

The procedure starts by finding, for each of the tutor syllables, the best-matched fragment in the pupil song as follows: For each tutor syllable (e.g. syllable ‘a’) the procedure finds the diagonal in the horizontal band of the similarity matrix (e.g. for syllable ‘a’) that maximizes the sum of similarity scores along it. One can think of this as ‘sliding’ a diagonal line over the horizontal band and finding the position at which the sum is maximal (red diagonal lines in Figure 3). The maximal sum defines a similarity score for each tutor syllable (termed ‘partial similarity score’). Because the sum is always computed over the whole diagonal, the highest partial similarity score is obtained when the fragment of pupil song matches the entire tutor syllable. Note that, if a short and long syllable are equally well imitated, the longer syllable is matched first.

Next, the procedure identifies the tutor syllable and the matching fragment of pupil song that have the highest partial similarity score (Figure 3A, red diagonal in syllable ‘d’, marked by a yellow circle). Once this selection is made, the similarity matrix is modified to prevent re-matching the identified pupil song fragment with any of the remaining tutor syllables in later iterations of the algorithm. Specifically, the regions of the similarity matrix corresponding to the matched tutor syllable and pupil song fragment are set to zero. In the example, matching syllable ‘d’ (Figure3A), results in zeroing the appropriate rows and columns, as shown in Figure3B.

Once the similarity matrix is updated, the steps above are iterated. The sum over all the diagonals is recomputed for all the remaining tutor syllables. The tutor syllable that has the largest diagonal sum (best match) is selected, and the similarity matrix is updated again. This process continues until each of the tutor syllables is matched to a pupil song fragment (Figure 3B–E). The overall acoustic similarity score is the sum of the partial similarity scores for all tutor syllables, normalized by the sum of the lengths of all syllables in the tutor motif. The details of this procedure are described in Methods.

### Sequence similarity score

The overall sequence similarity score between a tutor and pupil song is computed as the average of ‘partial sequence similarity scores’ for each of the tutor syllables. For each tutor syllable, the partial sequence similarity score is defined to be the acoustic similarity of the pupil song with the next tutor syllable. Tutor syllable ‘d’, for example, would receive a high partial sequence similarity score if, after a good imitation of syllable ‘d’, the pupil then immediately produces a good imitation of syllable ‘e’. In Figure 3F (top panel), the fragment of pupil song that best matches tutor song ‘d’ is identified (we refer to this fragment of pupil song as ‘d_pupil_’). The partial sequence score for tutor syllable ‘d’ is defined as the acoustic similarity between tutor syllable ‘e’ and the fragment of pupil song that immediately follows d_pupil_. This is computed from the maximal diagonal in an area of interest in the similarity matrix (dashed red rectangle). The boundaries of this area of interest are made broad enough to allow some flexibility in the precise alignment of the potential imitation of syllable ‘e’ (see Methods). This method produces a ‘soft’ punishment for pupil syllables that are sufficiently misaligned as to extend outside the area of interest. The step-by-step process for computing the sequence similarity score is described in Methods.

A composite measure of similarity, referred to as the *similarity index (SI)*, is defined as the product of the acoustic similarity score, described above, and the sequence similarity score.

### Selecting an optimal set of features

Because of the high degree of stereotypy of zebra finch song, an algorithm for evaluating of song similarity should clearly assign a high similarity between different renditions of songs from the same bird (‘self-similarity’). Likewise, the large diversity of zebra finch songs should result in a low similarity between songs of unrelated birds in the colony (‘cross-similarity’). We set out to optimize the parameters of our model with respect to the contrast between self-similarity and cross-similarity, as measured by our algorithm. Contrast is defined as the difference between these two measures, normalized by their sum (see Methods). This metric has the advantage that it is invariant to overall changes in the scale of the similarity scores. Ideally, the contrast should be as large as possible, up to its maximal value of one.

**Figure 4:**
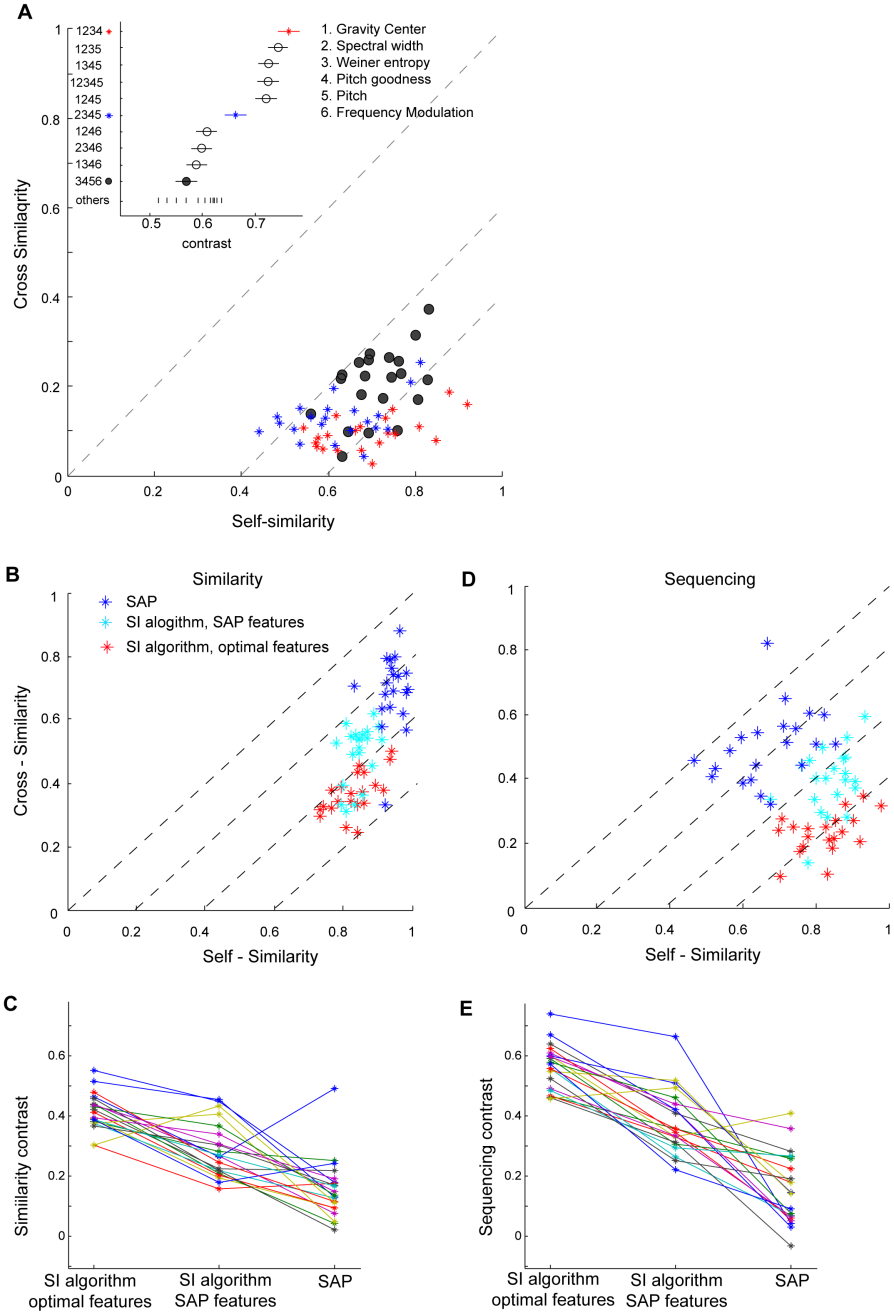
Comparison of different methods of measuring acoustic similarity. A) Selecting the optimal set of features. For each bird we measured the similarity of extracted song bouts to its own song motif (self-similarity) and to the motif of other birds (cross-similarity). These were computed using different combinations of spectral features (indicated with different colors and symbols). Inset: The contrast between self-similarity and cross-similarity, shown for each different subset of features tested. B-E) The SI algorithm yields higher contrast than the SAP software. Acoustic (B) and sequence (D) self-similarity versus cross-similarity computed using the Similarity Index (SI) algorithm with the optimal features (red), using the SI algorithm with the set of features used by SAP (cyan), and using SAP software (blue). The contrast was significantly larger using the SI algorithm with optimal features, both for the acoustic similarity scores and sequence similarity scores (C and E, respectively).

We set out to find a set of spectral features that maximally distinguishes between similar and dissimilar songs, according to the contrast metric. To do this, we tested the performance of our software with different subsets of features. All possible combinations of four, five or six spectral features out of the six described above were tested. For each combination of features we computed the self-similarity of multiple songs of the same bird and the cross-similarity of songs of unrelated birds (n = 21 adult birds). A self-similarity and cross-similarity scores were computed separately for each bird, using the Similarity Index (SI) computed by our algorithm (incorporating both acoustic and sequence similarities, see Methods). Figure 4A shows the detailed results of three of the feature combinations. This analysis suggested that some sets of features were more sensitive to the differences in the songs of different birds.

To quantify the effectiveness of different feature sets, we then computed the contrast between self-similarity and cross-similarity scores for each bird, as described above, and analyzed the distribution of contrast values obtained with different feature sets (see inset). A multiple comparison analysis (using the Tukey–Kramer method and 5% confidence) showed that sets of feature that included ‘frequency modulation (FM)’ performed significantly more poorly than other feature sets. In contrast, we found that feature sets that included ‘Gravity center’, but excluded FM, performed better than other sets. The best performance was obtained using the four parameters ‘Gravity center’, ‘Spectral width’ ‘Pitch goodness’ and ‘Weiner entropy’, but combinations that also included ‘Pitch’ performed nearly as well.

### Comparison to Sound Analysis Pro (SAP)

To further assess the performance of the SI algorithm, we compared the contrast scores obtained from SI with the contrast scores obtained from SAP. Since SAP software reports acoustic similarity and sequence similarity scores separately, we compare these separately in the following analysis (Figure 4B,C and Figure 4D,E for acoustic and sequence similarity, respectively, and Figure S1). Self- and cross-similarity scores used to compute contrast were obtained using the ‘similarity batch’ tool in SAP. We compared the contrast scores obtained from SAP to those obtained from the SI algorithm, first using the optimal set of features in the SI algorithm to compute the self- and cross-similarity scores. In this case, the contrast obtained with the SI algorithm (average acoustic similarity contrast = 0.41, red asterisks) was significantly higher than that reported by SAP (averaged acoustic similarity contrast  = 0.156, blue asterisks) for the same dataset (Figure 4B,C, paired t-test p<10^−6^,Wilcoxon rank-sum test, p<10^−5^). An even more profound difference was found when comparing the contrast in sequence scores (0.55 for SI compared to 0.173 for SAP paired t-test p<10^-7^,Wilcoxon rank-sum test, p<10^−7^). The comparisons described above were based on the contrast metric; similar findings were obtained for the difference between cross- and self- similarity scores (not normalized by the sum) (Figure S1).

To determine how much of this improvement in performance of the SI algorithm was due to the optimized feature set, we repeated this analysis using the SI algorithm with the same set of features used by SAP (Weiner entropy, frequency modulation, pitch and pitch goodness). Even when using the SAP feature set, the SI algorithm still reported a larger contrast metric (cyan asterisks, Figure 4B–E, acoustic similarity contrast  = 0.29, sequence similarity contrast  = 0.37, paired t-test, both p<0.001).

The analysis described above may have underestimated the performance of SAP. The contrast reported by the SAP algorithm (in comparing similar and non-similar songs) degrades when using pupil song segments that are longer than the tutor motif, since this results in an overestimate of similarity. (In the analysis above, both SAP and SI were forced to use the same data set, in which pupil segments were twice the length of the tutor motif.) Indeed, when the above analysis is run with pupil segments equal in length to the tutor motif (a more optimal configuration for SAP), both self- and cross-similarities are smaller, and the contrast increases significantly (comparison to data in Figure 1C–F black: Wilcoxon rank-sum test p = 0.04 and p = 0.02, paired t-test p = 0.003 and p = 0.001 for acoustic and sequence similarity, respectively). Even for this case, the SI algorithm produced a significantly higher contrast than SAP (p<10^-4^ for both acoustic and sequence contrast, Wilcoxon rank-sum test).

**Figure 5:**
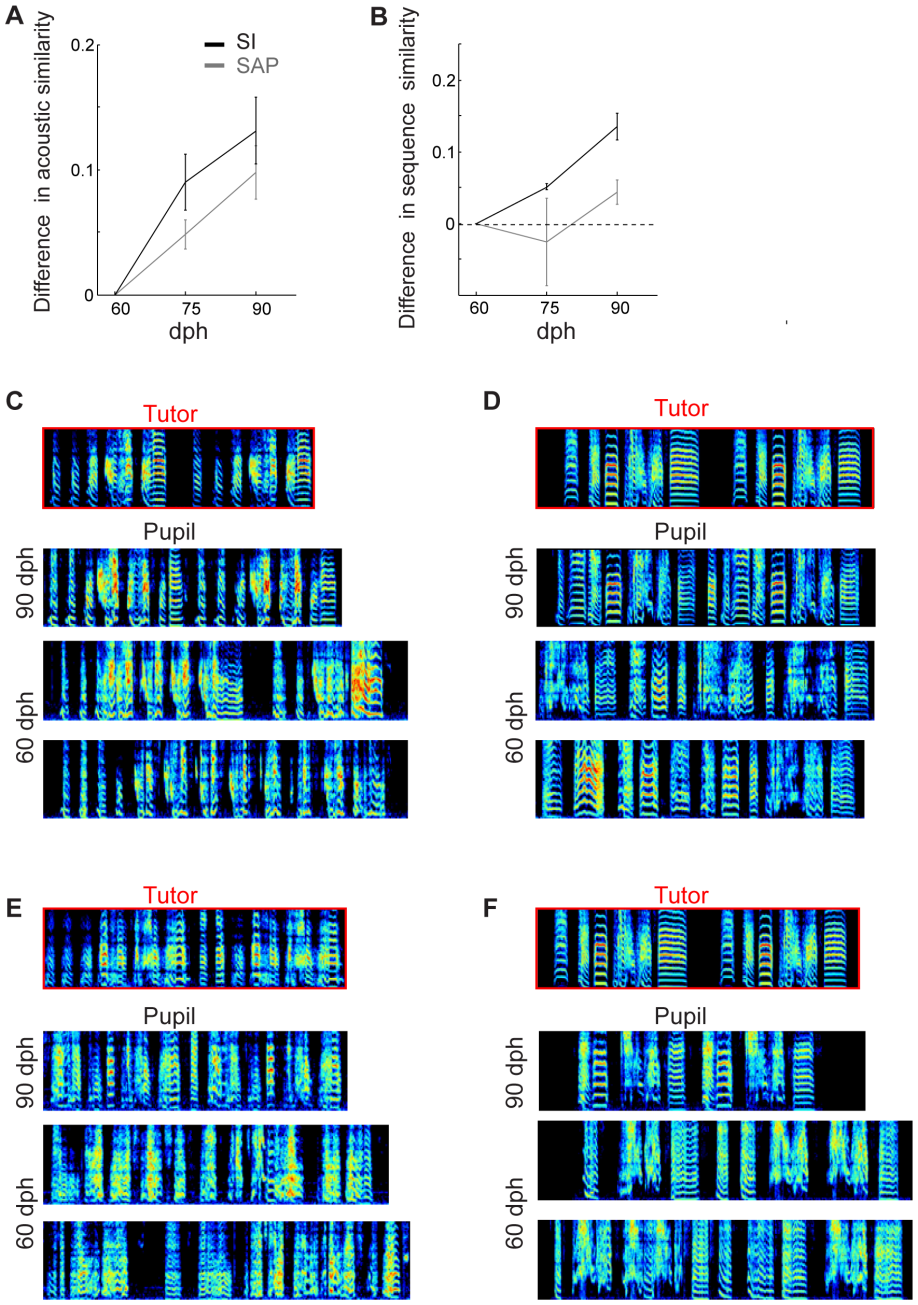
Changes in acoustic and sequence similarity through vocal learning. Quantification of tutor imitation in a set of four juvenile birds at early, middle and late stages of vocal learning. For each juvenile bird, song similarity to the tutor song was quantified on days corresponding to 60, 75 and 90 days of age (days post hatch, dph). Acoustic similarity and sequence similarity were quantified separately, and developmental changes were computed by subtracting the similarity scores at 60 dph. (A) Change in acoustic similarity at 75 and 90 dph. Shown are scores computed using the SI algorithm (black) and the SAP algorithm (grey), both of which show significant developmental increase in similarity to tutor song. B) Change in sequence similarity at 75 and 90 dph. The SI algorithm reveals significant development of sequence imitation. The SAP sequence scores exhibit no significant correlation with age. C–F) For each of the four birds songs early and late in development. Each figure corresponds to one bird top to bottom: tutor song; bird song recorded at age 90 dph; two examples of bird song recorded at age 60 dph.

Last, we demonstrate the performance of our algorithm in assessing the development of tutor imitation. We measured the extent of tutor imitation in young birds (n = 4, see Methods) at three stages of vocal development: at age 60 days post hatch (dph), when song imitation is in its early stages, at age 75, and at 90 dph, when song is relatively mature. Imitation was assessed by measuring the acoustic and sequence similarity between tutor song and juvenile song. Change in imitation scores over this period were determined by subtracting the scores at age 60 dph (Figure 5). Acoustic and sequence similarity scores were computed using both the SI algorithm, with automatically-selected pupil song segments, and using the SAP algorithm with manually-selected pupil song segments. Both the SI and SAP algorithms showed a consistent and significant increase in acoustic similarity during song development (Figure 5A, age 90 dph: t-test p = 0.016 and 0.02 for SI and SAP respectively). Note that the increase in acoustic similarity scores using SI was significantly larger than in SAP (paired t-test p = 0.03, 75 dph and 90 dph days combined). In terms of sequence similarity, the SI algorithm revealed a significant increase in sequence scores for all four birds between 60 and 90 dph (t-test p = 0.01), while SAP detected a significant increase in only two of the birds (t-test p<0.05). Furthermore, the SI algorithm revealed a significant trend in sequence scores across all four birds (p = 0.001), while SAP did not detect a significant overall trend (p = 0.43). Visual inspection of the songs revealed that all four birds exhibited improvements in sequence imitation during development (Figure 5C–F).

## Discussion

The quantification of song imitation is an important tool for understanding the mechanisms of vocal learning in songbirds. Advances in methods for analyzing the spectral structure of vocalizations [27] have recently led to an automated algorithm, available as a widely-used software package (Sound Analysis Pro; SAP), for computing the similarity of pupil and tutor songs [26]. In this method, a short (∼0.5–1 s) segment of pupil song and of tutor song are manually selected and passed to the program, which then computes a scalar measure of similarity. An overall similarity score is computed as the average over many of these comparisons across different segments of pupil and tutor song.

Selection of song segments for comparison poses a significant challenge for quantification of song imitation. If we imagine the simplest case in which both the tutor and pupil bird produce a perfectly stereotyped song motif, then selecting song segments would be trivial: simply manually extract a single example each of the tutor song motif and of pupil song motif. However, in laboratory studies of vocal learning, the pupil is typically a juvenile or young adult bird that may have undergone some experimental manipulation, and thus may have a quite variable song, perhaps even lacking a motif structure [24]. In contrast, the tutor is most often an adult bird with a fairly stereotyped song motif. In the procedure described in this paper, we have primarily addressed the problem of pupil song variability, and assumed that the tutor song is largely stereotyped. We will return later to the problem of tutor song variability.

Manual selection of pupil song segments can reasonably be used in the presence of small amounts of pupil song variability, but is problematic when the variability is large. If the pupil only rarely produced song variants, these might be safely ignored as ‘outliers’. Even if the pupil bird produced a small number of easily-identified motif variants, a manual selection process could still be used to choose the different variants for comparison. However, under conditions in which the pupil produces unreliable motifs, highly variable syllables or even syllables that have an acoustic structure not typical of zebra finches [24], [25], the process of manually selecting song segments could lead to an overestimate of song similarity, particularly if the selection process is biased toward ‘normal’ looking vocal sequences, as shown in Figure 1.

To avoid the need to hand-select segments of pupil song, the SI software automatically cuts continuous bouts of singing into equal-length segments for comparison to the tutor motif. Bouts of pupil song were extracted manually from recorded song files using Song-GUI. However, care was taken not to break up continuous streams of vocalization, even if the bout appeared to contain atypical vocalizations that were not obviously part of the motif. Only introductory notes, and calls that occurred outside of singing, were excluded. In principle, this process could be further automated.

We found that the songs of our adult tutor birds were relatively well-structured and, therefore, that variation in the motif, and its segmentation into syllables and gaps, can be captured by a few representative samples: thus, we used Song-GUI to manually select tutor song motifs. This may not always be the case however, and one can imagine automating the process of selecting tutor song segments, much like we have described for pupil songs. For example, they could be selected automatically by breaking up bouts of tutor singing into roughly one-second segments. It should be noted that our algorithm would still treat tutor song and pupil song in a fundamentally different way: tutor song is segmented into distinct syllables such that gaps are not analyzed, while pupil song is maintained as a continuous stream of sound.

While our procedure assumes that tutor songs contain only one motif, with perhaps a small number of minor variants, some adult zebra finches may have substantial variability in their motifs, or may even lack a highly stereotyped motif structure. Such variability can cause a problem for our algorithm, as we have described it. First, tutor song variability, captured by including multiple samples of different tutor songs in the comparison, will reduce the measure of song acoustic similarity, even when the bird makes a good copy of the tutor song. To see this, imagine using our algorithm to analyze the imitation of a hypothetical bird that sings three different motif variations - 1, 2, and 3. A pupil bird that perfectly imitates all three variations should ideally get a very high imitation score. However, our algorithm, as currently specified, will give this hypothetical bird a middling imitation score because it averages the similarity of matching motifs (1,1; 2,2, and 3,3) together with comparisons of non-matching motifs (1,2; 1,3; 2,3). Such concerns could be addressed by basing the overall imitation score not on the average of imitation scores across multiple comparisons of song segments, but rather on the 90^th^ percentile of imitation scores. In this way, the algorithm would be quantifying the ‘best match’, rather than the average match of the pupil song segments to the tutor.

In addition to specifying a procedure for selecting song segments, we have also modified the method of calculating the similarity of these segments to the tutor. First, because of the unreliability of segmenting highly variable song, pupil song segments are maintained in continuous form. In contrast, tutor songs are more stereotyped, and therefore more reliably segmented. Because we eliminated tutor gaps from the similarity calculation, our algorithm avoids problems inherent in computing the acoustic similarity between silent gaps. Furthermore, because pupil songs are not segmented into syllables and gaps, tutor song can be matched to the pupil song on the basis of entire syllables. This avoids introducing excessive flexibility in the assignment of regions of similarity, which can lead to an overestimate of the similarity of unrelated songs. The additional constraint imposed by the SI algorithm, of matching entire tutor syllables, also likely accounts for the increased sensitivity to changes in sequence similarity. This increased sensitivity was apparent in our quantification of song imitation during song development in juvenile birds.

Finally, we tested different combinations of features to find the set that performs best. Our objective assessment for performance was the contrast between similarities of bird song motif to its own song as compared to songs of unrelated adult birds. Optimally, the algorithm should differentiate well between these measures and produce high contrast. We included in our pool the features pitch, pitch goodness, wiener entropy and frequency modulation (FM), that are thought to bear a close relation to sound production (Ho et al. 1998). In addition, we added two new spectral features: gravity center and spectral width. The optimal subset of features included gravity center, spectral width, pitch goodness and wiener entropy, thus excluding pitch and FM. In our experience, these features seemed to be hard to estimate and tended to be unstable. SAP chose to expand the definition of pitch as both the frequency of the single tone (whistle) and the fundamental frequency (harmonic stack). SAP uses the Cepstrum [28] or the YIN [29] algorithm to detect harmonic pitch, and dynamically shift to gravity center when harmonic pitch is undetectable. However, many sounds are neither a harmonic stack nor a single tone. In these cases the pitch and FM are noisy signals that contribute little to the representation of the sound and can result in underestimation of similarity between such sounds. Indeed, including pitch and FM in our analysis resulted in favoring similarity between harmonic stacks over other sounds. We did not attempt to change the weights assigned to each acoustic feature and its contribution to the similarity. It is very much possible that a certain set of weights performs better (i.e. higher contrast). However, given the large space of possibilities we decided to simply give equal weights to measures from all features analyzed.

While our motivation in developing our algorithm was to assess the extent to which pupil bird has imitated the songs of its tutors, it can also be used to examine the stability of songs after a manipulation, such as deafening, that causes the gradual degradation of song acoustic and temporal structure. In this case, the song motif produced before the manipulation is treated as the ‘tutor’ motif, and songs produced on each day after the manipulation are treated as the pupil song.

Note that, while our procedure for quantifying song imitation captures one measure of syllable order, namely the sequence similarity, there are other aspects of song sequence it does not capture. For example, Scharff and Nottebohm (1991)[24] quantified sequence linearity and sequence consistency, which describe the transitions between different song syllables. These analyses require that individual pupil song syllables be segmented and labeled (‘a’, ‘b’, ‘c’, etc) in order to identify specific transitions. In principle, the SI algorithm could be extended to automate the labeling of pupil song fragments by their similarity to tutor song syllables, and the resulting labels used to compute the linearity and consistency scores.

## Conclusions

Starting from the algorithm described by Tchernichovski et al [26], we have made a number of modifications designed to assess song imitation in birds with poor imitation and high song variability. First, we have described an automated method for selecting segments of pupil song, thus reducing subjective bias. Second, we have described a new algorithm for computing the similarity between continuous streams of pupil song vocalizations with the tutor motif. We have also optimized the set of acoustic features to improve the contrast between comparisons of similar and dissimilar songs.

## Methods

### Subjects

Subjects were adult male zebra finches (120–350 days post hatch, dph). Birds were obtained from the Massachusetts Institute of Technology zebra finch breeding facility (Cambridge, Massachusetts). Animal care was carried out in accordance with guidelines of the National Institutes of Health guidelines and approved by the Massachusetts Institute of Technology Committee on Animal Care (protocol 0712-071-15).

*Song recording*: Zebra finches were placed singly in a cage within a sound-attenuating chamber. Songs were digitized and recorded using Aardvard Direct Pro 24/96 and SAP sound analysis recorder software.

### Song spectral features

Song spectral features were computed from the song spectrogram as follows: The sound was first band-passed between 500 and 8600 Hz. It was then sampled in 9 ms windows in 1 ms sliding steps. The spectrogram was computed using multitaper spectral analysis [30] (time-bandwidth product, p = 1.5; number of tapers, k = 2). The song spectral features were computed for each short time window of the spectrogram. Each feature used in our algorithm was first mean-subtracted and normalized by the standard deviation of the distribution of that feature, as measured from a sample of 100 different songs recorded from 10 different adult birds from our colony.

*Wiener entropy* (also known as spectral flatness): A measure of sound randomness in which the width and uniformity of the power spectrum are evaluated. By definition, it is a number between 0–1. However, we measure it in logarithmic scale to expand the range. Therefore, it ranges from zero, for white noise, to minus infinity, for complete order (such as a single tone).

*Frequency modulation*: A measure of the slope of the frequency contours. It is computed by the angle between the time and frequency derivatives of the song power across frequencies.

*Pitch* is the perceived tone of sounds and a measure of the period of the sound oscillations. For sounds with multiple harmonics, it is the fundamental frequency, which was evaluated using Fourier transform of the log spectrum, a method known as the cepstrum [28], as used by SAP.

*Pitch goodness* is a measure of how well the pitch is defined. It is computed by the power, in the cepstrum, of the pitch. Therefore, it is a good detector for sounds with multiple harmonics, for which the Fourier transform of the log power spectrum has a distinctive peak.

In addition to these standard SAP features, we considered two additional spectral features:

*Gravity center*: The power spectrum at each time point is a distribution of weights along the frequency axis. The gravity center is the unique frequency point where the weighted relative distance of the power spectrum sums to zero. This is the first moment of the power spectrum.

Let *b_i_*, *i* = 1,…,*B*, be the discrete frequency points in the spectrum, and *p_i_* the power at each frequency. Then the center of gravity
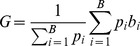

*Spectral width*: Measure of the extent to which the power is distributed around the gravity center. Mathematically, it is the second moment of the power spectrum. Following the notation above, the spectral width is computed as: 
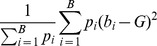



### Procedure for computing acoustic and sequence similarity score

#### Acoustic similarity score

The following contains a step-by-step description of the algorithm for computing the acoustic similarity score from the similarity matrix:

1. For each tutor syllable k (k = 1..N_syll_), find the fragment of pupil song that has the highest partial similarity to tutor syllable k. This is carried out in the following steps, where tutor syllable k starts at time bin I^k^_1_ and stops at time bin I^k^_2_ and has a length of N_k_ = I^k^_2_–I^k^_1_ bins:

a. Compute the partial similarity *S_P_^k^(j)* of tutor syllable k with each fragment of pupil song beginning at pupil song time bin *j* and ending at pupil song time bin *j+N_k_*. The sum of diagonals in the similarity matrix is given by: 
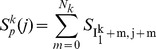



b. Find the bin j* for which the fragment of pupil song has the largest partial similarity S^k^_max_(j*) = 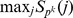. This says that tutor syllable k is best matched to the fragment of pupil song beginning at bin j* and extending to j*+N_k_. The partial similarity score of this match is S^k^_max_ =  S_P_^k^(j*).

2. From all the tutor syllables, choose the syllable k* with the highest partial similarity score.

3. Set rows I^k*^_1_ through I^k*^_2_, inclusive, to zero. The boundaries of the matched pupil fragment are j* and j*+N_k*_. Set the columns of the similarity matrix between these values, inclusive, to zero. The removal of these rows and columns ensures that, once a song segment is found to have a best match, it will not be matched again. Save the boundaries of the matched pupil fragment for the tutor syllable k* in the following vectors: J^k*^_1_ = j* and J^k*^_2_ = j*+N_k*_.

4. Return to step 1, discarding all previously calculated partial similarity scores. If a best match has been found for all syllables, then continue to the next step.

5. The final similarity score is a weighted average of partial similarities of all syllables.
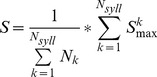



#### Sequence similarity score

Following the annotations above for each syllable k, we compute the similarity between tutor syllable k+1 and the fragment of pupil song between J^k^_2_ and J^k^_2_+ (I^k+1^_2_–I^k^_2_) +50 ms. Thus, the area of interest starts at the end of the pupil fragment matching syllable k. It has a length equal to 50 ms plus the interval between the offset of tutor syllable k and the offset of tutor syllable k+1. The additional 50 ms is intended to provide greater flexibility to the temporal alignment of sequential syllables. The sequencing score for syllable k is the maximal sum along a diagonal in the area of interest. Only the parts of the diagonals that are inside the area of interest are summed up. Therefore, different diagonals have different lengths according to their position relative to the borders of the area. Note that the partial sequence similarity score of a tutor syllable will not be computed if: 1) it is the last syllable in the tutor motif, or 2) it is matched to a pupil fragment too close to the end of the pupil song segment. The overall sequence score, 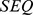, is the average over all the applicable syllable sequencing score.

### Calculation of self-similarity and cross-similarity

To assess the effects of different methods of sampling song segments and different acoustic features (Figure 1C–F and Figure 4), we computed the similarity between segments of song extracted from one bird and song motifs from the same bird (self-similarity), or song motifs from other birds (cross-similarity). Adult zebra finch songs can be highly stereotyped; thus comparisons between different songs of the same bird provide a natural means to quantify the performance of an algorithm at the upper bound of song imitation. In contrast, the songs of unrelated adult birds can be quite different, and provide a natural means to quantify the performance of an algorithm at the lower limits of song imitation.

Twenty-one unrelated adult birds from our colony were used for this analysis. For each bird, song motifs and song bouts were extracted and saved using Song-GUI. Details of the comparisons carried out for the different analyses of self-similarity and cross-similarity in each figure is explained below.

*Figure 1C*–*F*: For each bird in the database (n = 21 birds), 10 motifs were manually selected and roughly 20 song bouts were extracted, both using Song-Gui. For computing contrast between manually-selected song segments (blue), self-similarity was computed between all pairwise combinations of the 10 selected song motifs. For each bird, a set of 10 other birds was selected randomly for carrying out a cross-similarity comparison. For each point in Figure 1C–F, the cross-similarity scores were averaged over these 10 birds. For each cross-similarity comparison, the following steps were taken: For manual selection (blue), the comparisons were made between all pairwise combinations of the 10 manually-selected motifs using Sound Analysis Pro (SAP). For automatic selection (black), comparisons were made (using SAP) between all combinations of 10 manually-selected motifs and 25 motif-length segments, automatically extracted from the song bouts. For this calculation, the automatically extracted segments were chosen to be the duration of one song motif (rather than two song motifs described for the tutor-pupil imitation score). This was done to ensure that the manual/automatic comparisons were made on segments of the same length.

*Figure 4* *(all panels)*: Song bouts were saved using Song-GUI. Segments were extracted automatically by SI algorithm from within song bouts. Each song segment was twice the length of the song motif used in the comparison. For each set of features, and for each bird, self-similarity score was computed by randomly choosing 25 song segments and comparing them with randomly chosen 3 song motifs from the same bird, using SI algorithm. Cross-similarity was computed for each bird, by randomly choosing 25 song segments and comparing them with song motifs from 10 randomly selected birds.

### Contrast

We evaluated the performance of the SI algorithm and the SAP algorithm using two different metrics. First, we measured the contrast between the self-similarity and cross-similarity scores, defined as follows:
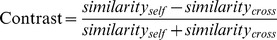
where the self- and cross-similarity scores were computed as described above. We also examined the difference between self- and cross-similarity scores as a metric of performance (Figure S1). We note, however, that the contrast metric has the advantage that it is invariant to overall scale. Thus, if two algorithms produce similarity scores that differ only by a constant factor, the contrast metric will indicate that the two algorithms have the same performance, while the difference metric will indicate that the algorithm with higher scores performs better.

### Tutor imitation through development

Male juvenile birds were maintained in the aviary in their home cages with both parents until age 44–45 days post hatch (dph), at which point they were transferred to isolated sound proof chambers where they were maintained. Songs were recorded continuously until they reached the age of 90 dph. Adult father (tutor) birds were transferred temporarily to sound isolation cages and their undirected songs were recorded. Acoustic and sequence similarities to the tutor song were measured using songs recorded on days corresponding to ages 60, 75, and 90 dph. At least 25 songs from each bird were used on each day. Comparisons of tutor motif to songs of juvenile birds were carried out using both the SI algorithm and using SAP. For SI algorithm, song bouts were extracted and comparisons to tutor motifs were computed using the Song-GUI software. For SAP analysis, motif-length song sections were manually extracted using Song-GUI and the sound segments were transferred to SAP for acoustic and sequence comparison. Changes in song similarity during development were determined by subtracting, for each bird, the score at age 60 dph from the scores at the later days. The analysis was carried out separately for acoustic similarity and sequence similarity scores.

## Supporting Information

Figure S1 **Comparing the performance of SI and SAP algorithms using the difference between self-similarity and cross-similarity.** SI algorithm with the optimal features (red), SI algorithm using with the set of features used by SAP (cyan), and SAP software (blue). The difference was significantly larger using SI algorithm with optimal features, both for (A) the acoustic similarity scores and (B) sequence similarity scores (Tukey–Kramer method with 5% confidence).
(TIF)

File S1 **Implementation of the SI algorithm in Matlab.** The software includes a manual and user friendly interface Song_gui.
(ZIP)
