# Correction: An Automated Procedure for Evaluating Song Imitation

**DOI:** 10.1371/journal.pone.0106169

**Published:** 2014-08-13

**Authors:** The PLOS ONE Staff

There is an error in the Supporting Information File S1. Please see the corrected file below.

## Supporting Information

File S1 **Implementation of the SI algorithm in Matlab.** The software includes a manual and user friendly interface Song_gui.
(ZIP)
