# Supplementary figures and images for: An Automated Procedure for Evaluating Song Imitation
Source: PLoS One. 2014 May 8;9(5):e96484. doi: 10.1371/journal.pone.0096484 (PMC4014513; doi:10.1371/journal.pone.0096484)

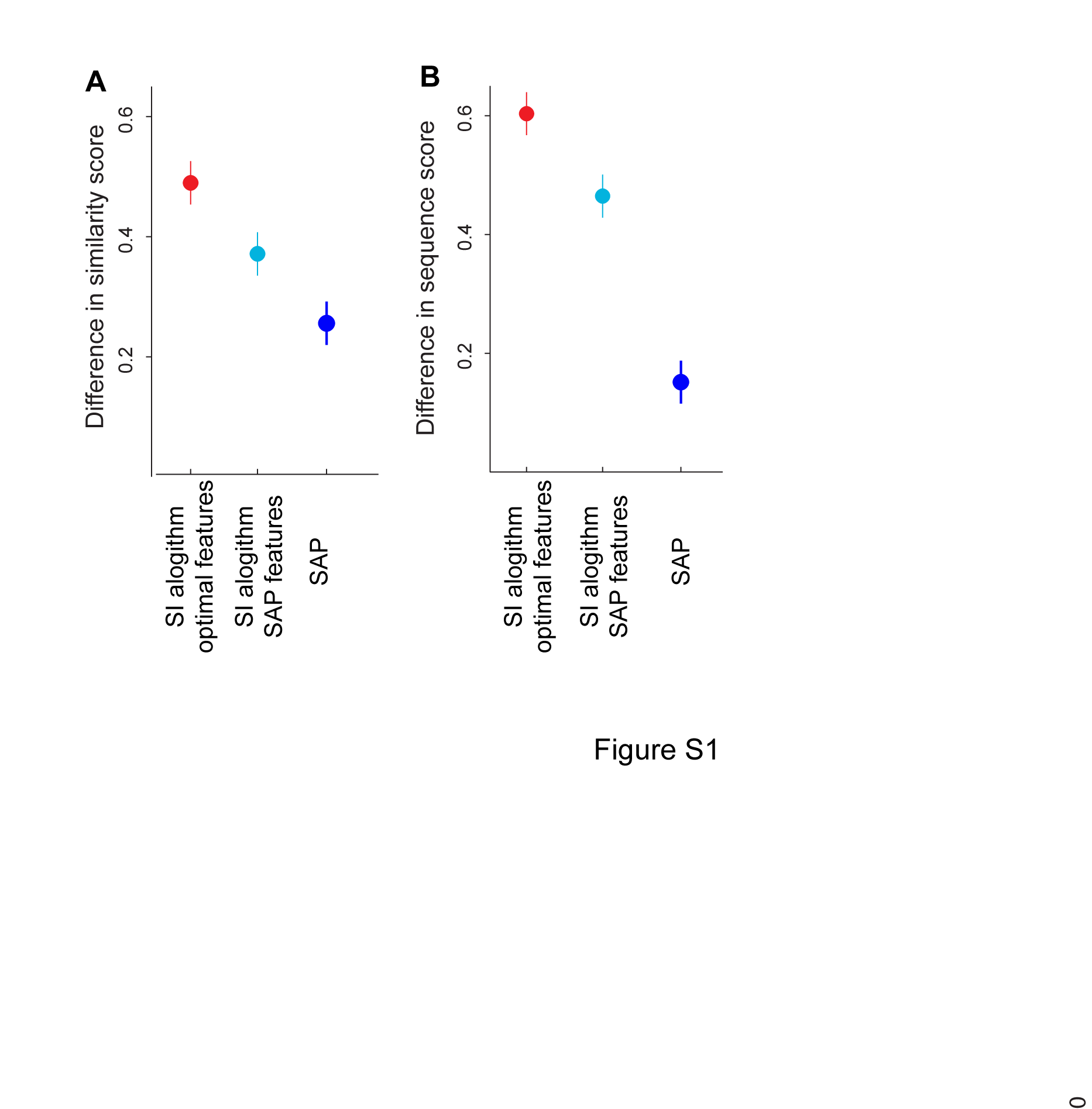

Supplement: Figure S1 — Comparing the performance of SI and SAP algorithms using the difference between self-similarity and cross-similarity. SI algorithm with the optimal features (red), SI algorithm using with the set of features used by SAP (cyan), and SAP software (blue). The difference was significantly larger using SI algorithm with optimal features, both for (A) the acoustic similarity scores and (B) sequence similarity scores (Tukey–Kramer method with 5% confidence). (TIF) [file pone.0096484.s001.tif]
