# Supplementary material for: An Automated Procedure for Evaluating Song Imitation
Source: PLoS One. 2014 May 8;9(5):e96484. doi: 10.1371/journal.pone.0096484 (PMC4014513; doi:10.1371/journal.pone.0096484)
Supplement: File S1 — Implementation of the SI algorithm in Matlab. The software includes a manual and user friendly interface Song_gui. (ZIP) [file pone.0096484.s002.zip › Similarity Index/Song_GUI manual.pdf]

# *The Similarity Index (SI) Software*

The SI software computes a metric of similarity between songs. The algorithm measures both acoustic similarity and sequence similarity, and generates a composite Similarity Index score that is a product of the two. Like some earlier methods for analyzing song similarity, the SI algorithm compares songs in a low-dimensional space of spectral features (Tchernichovski et al, 2000). However, the SI algorithm uses a different method of extracting song similarity, which was designed to compare stereotyped song motifs (for example, from a tutor bird) with continuous bouts of less-stereotyped song of another song (for example, from a young pupil bird). Finally, the SI algorithm uses a set of spectral features that were optimized for discriminating between similar and dissimilar zebra finch songs.

The SI algorithm computes acoustic and sequence similarity from a 'similarity matrix' that describes the acoustic similarity between the feature vectors (as a function of time) of the tutor song motif and a segment of pupil song. The segment of pupil song is automatically extracted from bouts of pupil song, and is chosen to be twice the duration of the tutor motif. For each segment of pupil song, the algorithm computes an acoustic and sequence similarity score. This calculation is repeated for many automatically-extracted segments of pupil song and for a small number (3-5) of renditions of the tutor motif. These comparisons result in a distribution of similarity scores, and the final acoustic and sequence similarity scores are given by the average of these distributions.

To facilitate the selection of song segments (which are input to the SI algorithm), we have provided a graphical user interface, called Song_GUI, that allows the user to extract tutor song motifs and pupil song bouts from directories containing raw .wav files. After the selection of song segments is complete, the SI algorithm can be run directly from the Song_GUI window. This document describes how to use the Song_GUI interface.

Song_GUI assumes that raw .wav files containing recordings of the tutor bird and pupil bird are saved in two separate directories. The program allows the user to automatically step through the files in these directories, to visualize the song spectrograms, to zoom in on song motifs or song bouts, and to export the selected regions as .wav files. The program also saves information identifying individual syllables in the tutor song, an important part of the similarity calculation. It also saves information about syllables in the pupil song, but this information is only used by the algorithm to aid in the automatic extraction of pupil song segments from the song file. The .wav files containing extracted song segments, and files containing ancillary information, are saved in a subdirectory within the directories containing the raw .wav files.

**<u>The Song_GUI window</u>**:

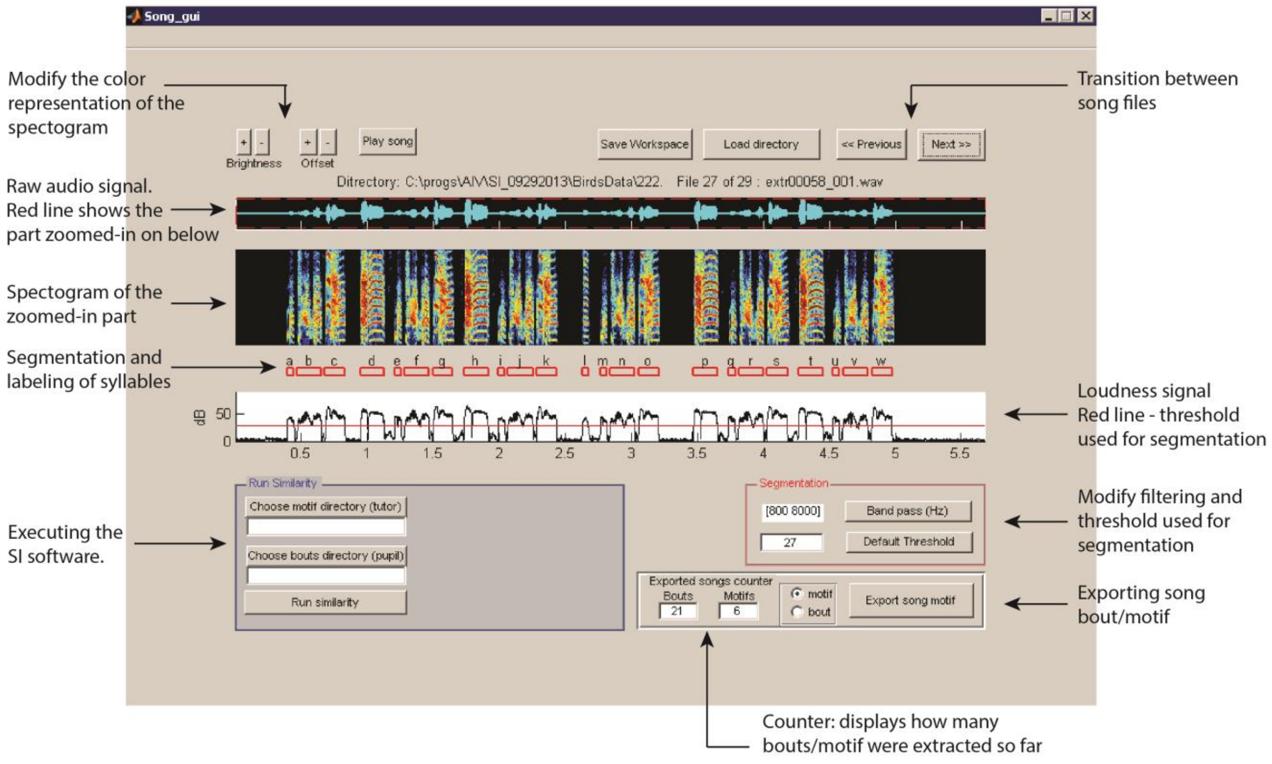

# Getting started

Download the software package to your computer and Run “Song_gui” from the command line in MATLAB.

## General guidelines:

***Song motifs (usually from tutor songs)***

- Click the ‘Load Directory’ button and choose the directory containing the tutor song .wav files (see section 1.1).
- Zoom in on song motifs and click the ‘Export song’ button (see section 2.1). The motif will automatically be stored in a subdirectory ‘motif’ within the directory of raw song files.
- You should choose enough motifs to represent all the variability in the motif and in its segmentation into syllables and gaps (usually 3-5 examples is enough).

***Song bouts (usually from pupil songs)***

- Click the ‘Load Directory’ button and choose the directory containing the pupil .wav files (see section 1.2).
- Extract song bouts by zooming in on them, or by deselecting all extraneous non-bout sounds (see section 2.2). Then click the ‘Export song’ button. Song bouts will automatically be stored in a subdirectory ‘bouts’ within the directory of song files.
- The more song bout renditions you select, the better your estimation of song imitation will be.

***Run the Similarity Index algorithm***

Insert the path for the tutor motif subdirectory and pupil bout subdirectory and press the ‘Run similarity’ button. Note that for computing self-similarity, you will need to extract both motifs and bouts from the same bird.

## Step-by-step

**1. Load directory:** There are two ways by which you can open a directory of song files:

a) Run Song_gui from the MATLAB command line. A dialog box will open that asks you to choose the directory containing .wav song files.

b) If the Song_gui window is already open, you can click the ‘Load directory’ button to open the dialog box.

In either case, if you previously processed files in this directory and saved your work (see Section 5 - Save workspace), then the program will automatically pull up the saved information and present the file you last processed.

**2. Extract song motif/bout:**

Export song function will save, as a .wav file, the part of the song currently displayed in the spectrogram window. It will also save, in a text file, the segmentation information, including the threshold level, the syllable onset-offest times, and a binary indicating if they are selected or

deselected (0 or 1, respectively). The segments are saved in the same motif/bouts subdirectory that contains the extracted .wav files.

The file name provides information about where the song segment was extracted. For example, assuming segments are extracted from file name OriginalFileName. For example:
'bouts_sylls_a_g_OriginalFileName' - contains a bout extracted from the file OriginalFileName which includes syllables a,b,c,d,e,f,g.

'motif_sylls_b_e_OriginalFileName' – contains a motif extracted from OriginalFileName, which includes syllables labeled b,c,d,e.

**2.1. Song motif**:

Marking a rectangle on the spectrogram (in this example), or on the raw signal.

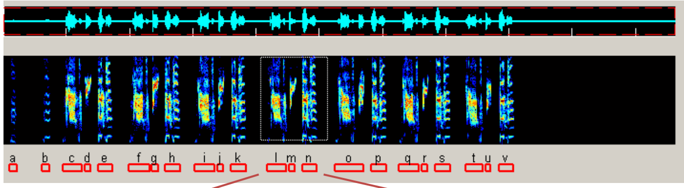

The spectrogram is zoomed-in. The zoomed-in part is marked by a dashed red rectangle on the raw signal.

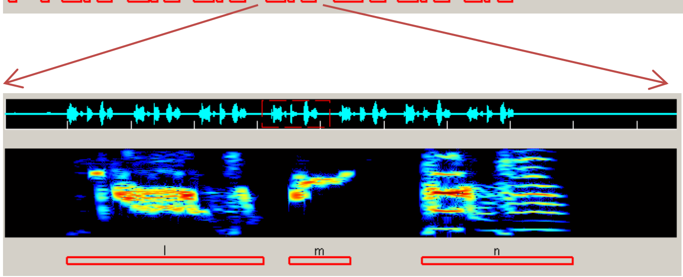


First, select a motif song by zooming in on it: Mark the borders by dragging the mouse (while holding the left mouse button) over the part of the spectrogram or raw signal you want to zoom in on.

Second, fix segmentation if needed, such that syllables are detected properly. The current segmentation is plotted by the red boxes below the spectrogram. Details how to modify the segmentation are given in section 3. Note that a reliable segmentation of motif songs is very important for the SI software.

Third, make sure the 'motif' button is selected in the Export box. Press the 'Export song motif' button. Note that the counter of exported song motifs increased by one.

Finally, if you want to zoom out to select another motif, double click the mouse on the spectrogram. If you want to load the next .wav file, click on the 'Next' button to proceed to the next file.

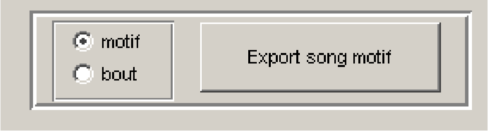


**2.2 Song bout**: Load the directory containing pupil .wav files. You want to extract bouts of singing, while removing contamination from introductory notes, calls, or other non-

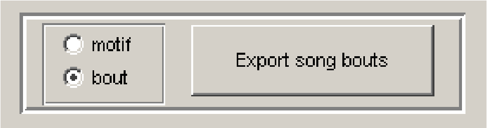

vocal contamination. If the song file you load contains only a single bout of singing, all you need to do is deselect segmented sounds that appear *outside* the song bout (see section 4). Then, press 'Export song bout'. Note that the counter of exported song bouts increased by one.

If the song file you are working on is composed of several bouts, one approach is to zoom in on each bout separately, as described above, and export each bout separately. An alternative approach, which may be less prone to experimenter bias, is to clean up the entire file, using the deselect function to eliminate any introductory notes and any calls or non-vocal sounds that occurr between bouts. Then export the entire song file using the 'Export song bout' button).

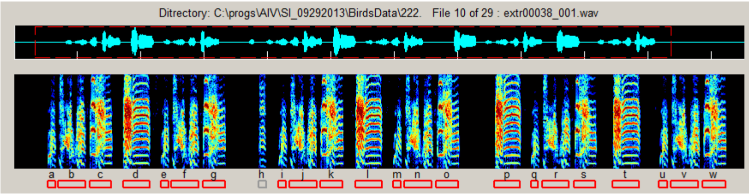


**Note**:

- De-selecting syllables in the middle of song bouts, just because they are unreliable or look atypical, may introduce experimenter bias. In short, we recommend keeping bouts of singing as intact as possible.

- The SI algorithm uses the segmentation of the pupil song only to determine when song bouts begin and end (for the purpose of automatically extracting song segments for analysis). Careful refinement of the segmentation is not necessary.

*Delete an extracted song bout/motif:* In case you made a mistake and you wish to delete an extracted song bout or song motif, you need to access the directory directly and remove the undesired .wav file along with the text file (and optionally also a .mat file) that has the same name. These files are located in the 'motif' or 'bouts' subdirectory within the directory containing the raw song files currently being processed.

## 3. <u>Threshold and segmentation manipulation:</u>

The segmentation control box allows adjustment of the bandpass filter use for computing computing the loudness signal and the current threshold that is used for segmentation.

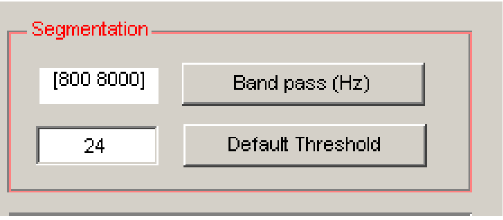


When switching to a new song file, the filter and threshold parameters are not changed from those used in the previous file. You can modify the threshold by (1) writing the desired level in the designated box or by a clicking the cursor in the loudness window. You can always return to the default threshold by pressing 'Default Threshold'. The current filter and threshold parameters will be maintained after you advance to the next file by pressing the 'Next' button.

If you change the parameters, and then switch back to a file that was processed earlier, the filtering and threshold parameters saved during the previous processing of that file will be reloaded.

Band pass (Hz) button: For certain songs you may need to modify the band pass limits. This will modify the loudness signal and consequently the syllable/gap segmentation.

**4. Select/deselect a syllable segment**

The segmentation line indicates possible syllables by red boxes and identifying letters. By default, all segments are considered eligible syllables and are therefore highlighted (boxes have red outlines).

There are two ways to deselect a highlighted syllable segment:

1. Using the keyboard: Type the range of letters corresponding to the segments you wish to deselect. For example, if you want to omit segments a,b,c and d, then type on the keyboard ‘a’ and then ‘d’. To deselect a single segment type its letter twice. Note: Before using the keyboard to select/deselect segments, you need to mouse click anywhere on the grey background area of the GUI.
2. Using the mouse: Drag the mouse around the red syllable segment boxes you want to deselect (or reselect).

Deselected segments are now marked by grey boxes in the segmentation line and will not be considered as song syllables for the similarity analysis. To reselect segments, perform the keyboard or mouse actions again.

**5. Save workspace:** This will save the segmentation information from all the files you went through – the threshold, segmentation, selected/deselected syllables.

**6. Run Similarity:** The steps for performing the similarity analysis are as follows:

For each of the .wav files in the bout and motif subdirectories, the program computes the timeseries of each spectral feature. The matrix of features as a function of time is saved as a .mat file (using the same filename as the source .wav file) in the same subdirectory. Note: This part may take some time. The more bouts you extract the longer it will take. The algorithm then continues as follows:

- The algorithm then cuts the song bouts into non-overlapping cut segments, each twice the length of the selected tutor motif.
- Compare between all song cuts and all tutor motifs.
- For each comparison the software calculates acoustic similarity, sequence similarity, and the composite similarity index (SI) scores, which is the product of acoustic and sequence similarity scores.

The output of the Similarity Index calculation:

- The output is shown in the Song_gui 'Run Similarity' window. It will show the histogram of the computed imitation scores, as well as the average.
- The program also generates an Excel spreadsheet file containing 3 columns of data: the acoustic similarity, the sequence similarity and the composite similarity index scores) with all the comparisons that were made in this run. The file is saved in the pupil bouts subdirectory. The name of the file is a concatenation of the date, tutor and pupil names: `filename=['SI_' date '_Motif_' TutorName '_Bouts_' BirdName];` The results are also saved in a .mat file containing all the same information as the Excel file.

**7.** **Examples**: We provide song files recorded from three birds under the subdirectory 'SongBirds':

- A pupil songbird (subdirectory: 'pupil1'). The bird only partially imitated its tutor.
- Two tutors (subdirectories 'Tutor1', 'Tutor2'). Note that Tutor1 was the tutor of pupil1.
